# A Novel Pattern Recognition Method for Non-Destructive and Accurate Origin Identification of Food and Medicine Homologous Substances with Portable Near-Infrared Spectroscopy

**DOI:** 10.3390/molecules30173565

**Published:** 2025-08-30

**Authors:** Wei Liu, Ziqin Zhang, Yang Liu, Liwen Jiang, Pao Li, Wei Fan

**Affiliations:** 1 College of Food Science and Technology, Hunan Agricultural University, Changsha 410128, China; liu12222020@126.com (W.L.); zzq15377425360@126.com (Z.Z.); fs.ly@hunau.edu.cn (Y.L.); hnndjlw@163.com (L.J.); 2 Guangdong Provincial Key Laboratory of Utilization and Conservation of Food and Medicinal Resources in Northern Region, Shaoguan University, Shaoguan 512005, China

**Keywords:** food and medicine homologous substances, origin identification, near-infrared spectroscopy, boosting–partial least squares–discriminant analysis, partial least squares–discriminant analysis

## Abstract

In this study, a novel pattern recognition method named boosting–partial least squares–discriminant analysis (Boosting-PLS-DA) was developed for the non-destructive and accurate origin identification of food and medicine homologous substances (FMHSs). Taking *Gastrodia elata*, *Aurantii Fructus Immaturus*, and *Angelica dahurica* as examples, spectra of FMHSs from different origins were obtained by portable near-infrared (NIR) spectroscopy without destroying the samples. The identification models were developed with Boosting-PLS-DA, compared with principal component analysis (PCA) and partial least squares–discriminant analysis (PLS-DA) models. The model performances were evaluated using the validation set and an external validation set obtained one month later. The results showed that the Boosting-PLS-DA method can obtain the best results. For the analysis of *Aurantii Fructus Immaturus* and *Angelica dahurica*, 100% accuracies of the validation sets and external validation sets were obtained using Boosting-PLS-DA models. For the analysis of *Gastrodia elata*, Boosting-PLS-DA models showed significant improvements in external validation set accuracies compared to PLS-DA, reducing the risk of overfitting. Boosting-PLS-DA method combines the high robustness of ensemble learning with the strong discriminative capability of discriminant analysis. The generalizability will be further validated with a sufficiently large external validation set and more types of FMHSs.

## 1. Introduction

At present, there are over one hundred food and medicine homologous substances (FMHSs), including *Gastrodia elata*, *Aurantii Fructus Immaturus*, and *Angelica dahurica*. The substances are rich in bioactive compounds, such as polyphenols and polysaccharides, which provide dietary benefits while naturally supporting immunity, metabolism, and cellular protection [1]. Even for the same FMHS, samples from different origins may have significant differences in chemical composition and content, which will directly affect their function and activity [2]. To obtain excessive profits, unscrupulous merchants may sell low-quality and low-priced FMHSs as high-quality products, seriously damaging the interests of consumers. Therefore, it is of great significance to establish an accurate method for the origin identification of FMHSs. At present, many technologies have been applied for the origin identification of FMHSs, including stable isotope ratio technology [3,4], mineral element analysis technology [5,6], chromatography technology [7,8], and chromatography–mass spectrometry technology [9,10]. Both stable isotope ratio and mineral element analysis techniques suffer from limitations, including matrix interference and database dependency. Although chromatography and chromatography–mass spectrometry have been used for the qualitative and quantitative analysis of the organic components in FMHSs, these methods still have some shortcomings, such as complex sample pretreatment, a long detection time, expensive equipment, and the need for professional personnel. In addition, the above methods are often destructive to samples. Near-infrared spectroscopy (NIR) technology can obtain the information on absorption characteristics of overtone and combination bands from hydrogen-containing groups in samples, enabling rapid, non-destructive, and convenient analysis of complex samples [11,12]. In the existing studies, NIR spectroscopy has been widely used in the origin tracing [13,14,15], adulteration identification [16,17,18], and component analysis [19,20,21] of FMHSs.

The interferences of peak overlapping, baseline drift, and signal noise are inevitable in NIR spectra, and it is necessary to use chemometric methods to analyze the complex spectra. A series of spectral pretreatment methods has been developed, which can eliminate various interferences in NIR spectra [22]. In order to achieve the origin identification, various pattern recognition methods were applied to establish the identification models. As the most common unsupervised pattern recognition method, the principal component analysis (PCA) method can effectively achieve dimensionality reduction [23]. Partial least squares–discriminant analysis (PLS-DA) is a common supervised pattern recognition method that combines partial least squares regression and discriminant analysis. The method focuses on finding features that maximize category differences during dimensionality reduction, holding better identification ability than unsupervised pattern recognition [24]. However, this method may easily lead to overfitting. As an ensemble learning method, the boosting strategy iteratively adjusts the sample weights based on the previous classification results and finally obtains the results by weighted average [25]. There were many studies on the boosting strategy to obtain more accurate quantitative prediction results [26,27,28]. The prediction of lime acidity was achieved using Boosting-PLS and NIR spectroscopy [28]. The correlation coefficients of pH and total acidity with Boosting-PLS were 0.84 and 0.66, while those of PLS were 0.82 and 0.65. However, to our knowledge, the Boosting-PLS method is mainly used to improve the predictive accuracy and robustness of quantitative models at present. There is still little research on the use of the boosting strategy for the identification analysis. Discriminant analysis (DA) is a statistical strategy that uses category information from datasets to create discriminant boundaries for classification [29]. Similarly to the principle of the PLS-DA method, a novel pattern recognition method can be developed by combining the Boosting-PLS and DA.

In this study, a novel pattern recognition method named Boosting-PLS-DA was developed to establish the origin identification models of FMHSs, compared with two existing pattern recognition methods (PCA and PLS-DA). The NIR spectra of *Gastrodia elata*, *Aurantii Fructus Immaturus*, and *Angelica dahurica* from different origins were obtained by portable NIR spectroscopy without destroying the samples. The models were evaluated using the validation set and an external validation set obtained one month later.

## 2. Results

### 2.1. Spectra of FMHSs

**Figure 1:**
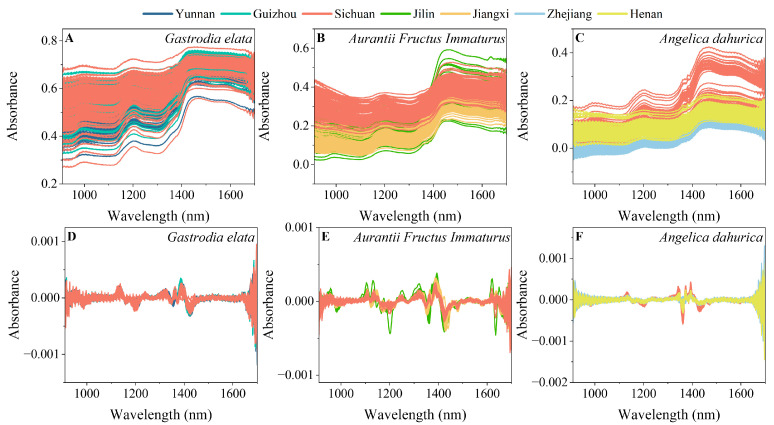
Original spectra of *Gastrodia elata* (**A**), *Aurantii Fructus Immaturus* (**B**), and *Angelica dahurica* (**C**) from different origins. Spectra with second derivative pretreatment of *Gastrodia elata* (**D**), *Aurantii Fructus Immaturus* (**E**), and *Angelica dahurica* (**F**) from different origins.

Figure 1A–C showed the original spectra of *Gastrodia elata*, *Aurantii Fructus Im-maturus,* and *Angelica dahurica*, respectively. As shown in the figures, absorption peaks were similar for all samples, which were located at 993 nm, 1200 nm, and 1446 nm. These variables were assigned to the O-H second overtone band, the C-H second overtone band, and the O-H first overtone band, respectively, which may belong to the absorption of water and carbohydrates. In addition, there was a certain difference in the spectra of *Angelica dahurica* from Sichuan compared to those from other origins within the range of 1400 nm to 1600 nm, while there was no significant difference in the spectra of *Gastrodia elata* and *Aurantii Fructus Immaturus* samples from different origins. It was difficult to accurately identify the FMHS’s origins based on the original spectra.

In addition, there were often severe interferences, such as background noise, baseline drift, and peak overlapping, in the NIR spectra. Six spectral pretreatment methods (standard normal variate (SNV) transformation, multiplicative scatter correction (MSC), first-order derivative (first derivative), second-order derivative (second derivative), continuous wavelet transform (CWT), and detrend correction (DT)) were used to eliminate the above interferences. The spectra with second derivative pretreatment of *Gastrodia elata*, *Aurantii Fructus Immaturus*, and *Angelica dahurica* are shown in Figure 1D–F. It could be seen that the background noise and baseline drift interferences can be effectively eliminated by the second derivative pretreatment, and the characteristic peaks can be made more prominent. Taking *Angelica dahurica* as a sample, the variables were located in the ranges of 1111–1144 nm and 1326–1408 nm, which may belong to the second overtone band of C-H and the first overtone band of O-H, respectively. However, there was significant noise interference above 1600 nm and below 1000 nm with the second derivative pretreatment for the three kinds of FMHSs. In addition, there were significant differences in the spectra of *Angelica dahurica* from Sichuan compared to those from other origins within the range of 1300 nm to 1450 nm, shown in Figure 1F. It was still difficult to accurately identify the *Gastrodia elata* and *Aurantii Fructus Immaturus* samples from different origins, even with the optimized pretreatment methods.

### 2.2. Identification Results of PCA

**Figure 2:**
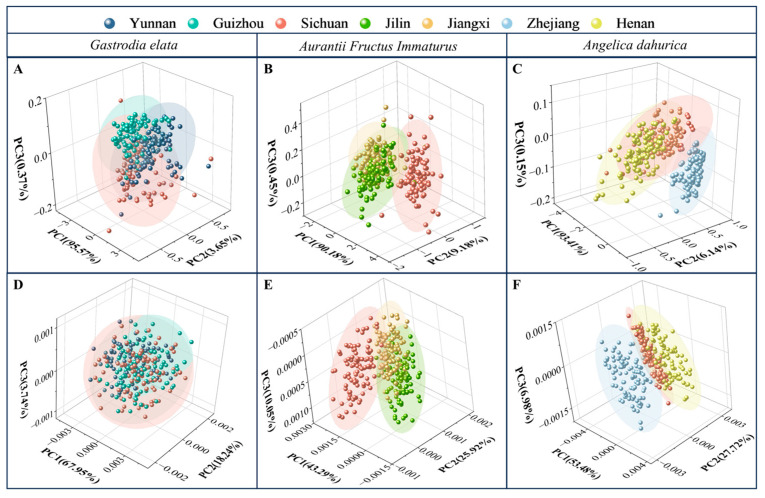
PCA results of *Gastrodia elata* (**A**), *Aurantii Fructus Immaturus* (**B**), and *Angelica dahurica* (**C**) with original spectra. PCA results of *Gastrodia elata* (**D**), *Aurantii Fructus Immaturus* (**E**), and *Angelica dahurica* (**F**) with second derivative pretreatment.

As the most common unsupervised pattern recognition method, PCA was applied to establish the model for origin identification of FMHSs. The first three scores (PC1, PC2, and PC3) were used, and the variance contribution rates were marked on the axes. The PCA results were shown in Figure 2A–C. As shown in the figures, for the three kinds of FMHSs, there were significant overlaps among data points from different origins. Six spectral pretreatment methods were used, combined with the PCA method. The PCA results with the second derivative pretreatment are shown in Figure 2D–F. Compared to the PCA model of original spectra, there was no significant improvement for the second derivative–PCA model, as shown in the figures. Although there were slight differences in the spectra after pretreatment, the PCA results were still unsatisfactory. Therefore, it was difficult to accurately identify the FMHS’s origins with the PCA method.

### 2.3. Identification Results of PLS-DA and Boosting-PLS-DA

**Figure 3:**
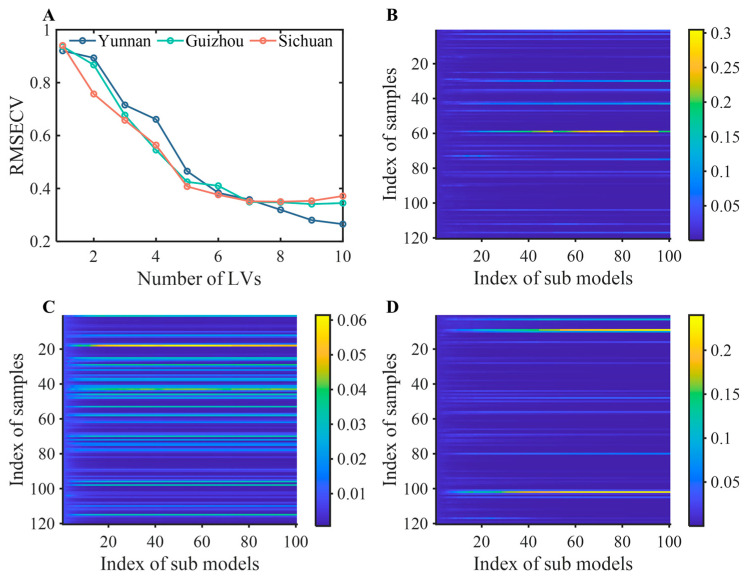
RMSECV values with the number of LVs for *Gastrodia elata* (**A**). Weights of selected samples on sub-models for *Gastrodia elata* from Yunnan, Guizhou, and Sichuan (**B**–**D**).

In this study, a novel pattern recognition method named Boosting-PLS-DA was developed to establish the origin identification models of FMHSs, compared with PLS-DA. Taking *Gastrodia elata* as an example, the variation in root mean square error of cross-validation (RMSECV) values with the number of LVs was shown in Figure 3A. The figure showed that the optimal number of LVs for the three origins was 7. The optimal numbers of LVs for *Aurantii Fructus Immaturus* and *Angelica dahurica* were obtained using the same strategy.

Boosting-PLS-DA was carried out by a weighted average of the output results from multiple PLS-DA sub-models to obtain the final prediction result. Taking *Gastrodia elata* as an example, Figure 3B–D showed the weights of selected samples in sub-models of Yunnan, Guizhou, and Sichuan, respectively. The different shades of colors revealed the weight size. As shown in the figures, the weights of the selected samples used to establish the sub-models for the three origins were different. There were high weights of sample 59 for Yunnan; sample 18 for Guizhou; and samples 9, 101, 102, and 103 for Sichuan. According to the principle of Boosting-PLS-DA calculation, the samples with high weights should be the misclassified samples. Additionally, multiple PLS-DA sub-models can reduce the impact of redundant variables weakly correlated with categories, further reducing the risk of overfitting.

**Figure 4:**
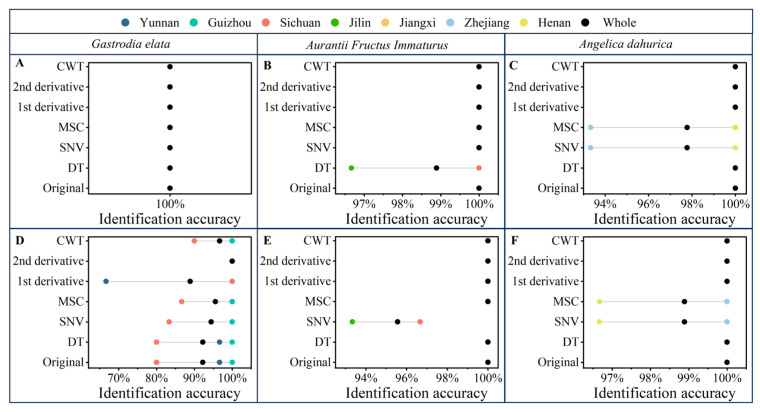
Identification accuracies of the validation sets (**A**–**C**) and external validation sets (**D**–**F**) with PLS-DA and pretreatment methods.

The Boosting-PLS-DA and PLS-DA models were evaluated using the validation set and an external validation set obtained one month later. The identification accuracies of the validation and external validation sets with PLS-DA and pretreatment methods are shown in Figure 4. Due to the utilization of prior knowledge, the identification results of PLS-DA were significantly better than those of PCA. For the original spectra of *Aurantii Fructus Immaturus* and *Angelica dahurica*, the PLS-DA identification results of the validation and external validation sets were 100%. However, for the original spectra of *Gastrodia elata*, the PLS-DA identification results of the validation and external validation sets were 100% and 92.22%, respectively. This is because the external validation sets were obtained one month later, and the distribution of the external validation sets may be different from that of the calibration and validation sets. It may lead to worse results for the external validation sets than for the validation sets. The same problem can also be found in the *Aurantii Fructus Immaturus*-SNV-PLS-DA model (100% for the validation set and 95.56% for the external validation set, respectively). Therefore, the PLS-DA models may have the risk of overfitting.

**Figure 5:**
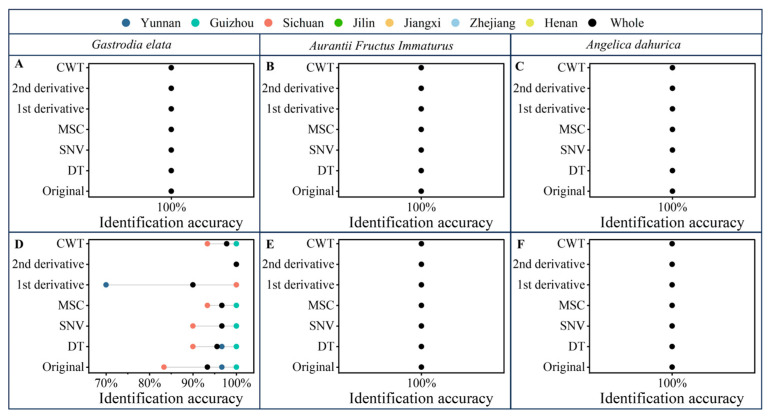
Identification accuracies of the validation sets (**A**–**C**) and external validation sets (**D**–**F**) with Boosting-PLS-DA and pretreatment methods.

The identification accuracies of the validation and external validation sets with Boosting-PLS-DA and pretreatment methods are shown in Figure 5. For the original spectra of *Aurantii Fructus Immaturus* and *Angelica dahurica*, the Boosting-PLS-DA identification results of the validation and external validation sets were 100%. For *Gastrodia elata*, the identification results of Boosting-PLS-DA (100% for the validation set and 93.33% for the external validation set, respectively) were better than those of PLS-DA. In addition, as seen in Figure 5A,D, the external validation set results based on the Boosting-PLS-DA method had been significantly improved compared with the PLS-DA method. Furthermore, for both *Aurantii Fructus Immaturus* and *Angelica dahurica*, the Boosting-PLS-DA identification accuracies of the validation sets and external validation sets with the original spectra and all spectral pretreatments were all 100%, which were significantly better than those of PLS-DA. The Boosting-PLS-DA method combines the benefits of high accuracy and robustness, which can be applied for the origin identification of FMHSs.

**Figure 6:**
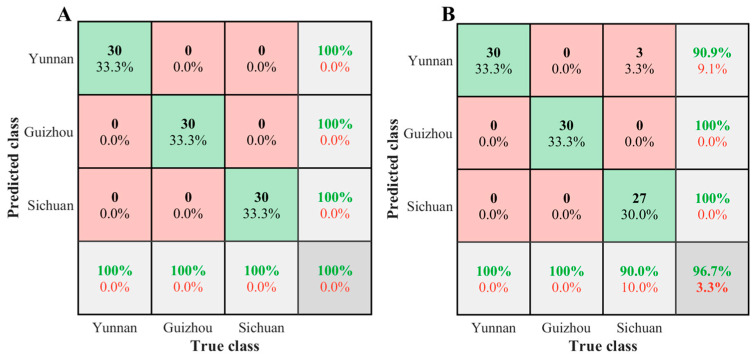
Confusion matrices of the validation set (**A**) and external validation set (**B**) for the *Gastrodia elata*-SNV-Boosting-PLS-DA model.

In order to further evaluate the performance of the Boosting-PLS-DA model, the identification results were visualized using confusion matrices. Taking *Gastrodia elata* as an example, Figure 6A,B showed the identification results of the SNV-Boosting-PLS-DA model. For the validation sets, the Boosting-PLS-DA method could achieve a 100% identification accuracy of FMHSs from different origins. In addition, for the external validation sets, only three samples from Sichuan were mistakenly identified as those from Yunnan, with an identification accuracy of 96.70%, which was better than the SNV-PLS-DA model (94.44%). In conclusion, the Boosting-PLS-DA method has higher robustness and accuracy than the PLS-DA method.

## 3. Discussion

PLS-DA algorithm is one of the most commonly used pattern recognition methods at the current stage and has been successfully applied for the identification analysis of various FMHSs. However, too many variables may lead to multicollinearity, and the problem of overfitting may occur when the number of variables is greater than the number of samples [30]. *Citri Reticulatae Pericarpium* and *Gastrodia elata* are common types of FMHSs. In previous studies, the mold-damaged *Citri Reticulatae Pericarpium* samples were identified using PLS-DA and NIR spectroscopy. However, the whole discrimination accuracy of the validation set was 100%, while that of the external validation set was only 89% [22]. For the variety identification of *Gastrodia elata* Blume, the PLS-DA identification accuracies of the validation and external validation sets were not the same (99.3% and 97.8%, respectively) [31]. All indicated that PLS-DA may easily lead to overfitting.

The boosting strategy can effectively reduce bias and variance by integrating multiple predictions of sub-models [32]. Terahertz (THz) spectroscopy coupled with PLS and Boosting-PLS was employed to analyze the water content in rapeseed leaves. Boosting-PLS model achieved more accurate quantitative prediction results than the PLS model. The R of calibration set obtained with Boosting-PLS was 0.8475, while that with PLS was 0.8387 [33]. In addition, the performance of Boosting–kernel PLS (Boosting-KPLS) was also superior to that of KPLS for the analysis of total nitrogen contents in dried tobacco leaves and Thai fish sauces [26]. The Rs of the prediction set of Boosting-KPLS and KPLS were 0.93935 and 0.93696, respectively, while the root mean square errors of prediction set (RMSEPs) were 0.07951% and 0.08093%, respectively, for the dried tobacco leaves. For Thai fish sauces, the Rs of the prediction set of Boosting-KPLS and KPLS were 0.99079 and 0.99004, respectively, while the RMSEPs were 0.12226% and 0.12818%, respectively. There was little research on the use of the boosting strategy for identification analyses. However, the shortcomings in adapting to classification tasks are inevitable in Boosting-PLS, and it is necessary to combine it with the DA method to achieve accurate category analysis.

NIR spectroscopy and many pattern recognition methods have been used for the origin identification of various FMHSs. NIR spectroscopy and three-dimensional correlation spectra–residual convolutional neural network (3DCOS-ResNet) were used for the origin identification of *Gastrodia elata* [34]. The accuracies of validation and external validation sets were 100% and 95.45%, respectively. The identification accuracy of the external validation set needs further improvement. For the origin identification of *Angelica dahurica*, the 94.7% accuracy was obtained with a data-enhanced convolutional neural network (CNN) algorithm and NIR spectroscopy [35]. No external validation set results were reported in this study. In addition, NIR spectroscopy research on *Aurantii Fructus Immaturus* mainly focused on the component analysis. The quantitative analysis of an ethanol extract in *Aurantii Fructus Immaturus* was achieved using NIR spectroscopy and the PLS method [36]. There was little research on the origin identification of *Aurantii Fructus Immaturus* with NIR spectroscopy. Similarly to the principle of the PLS-DA method, a novel pattern recognition method can be developed by combining Boosting-PLS and DA.

In this study, a new pattern recognition method named Boosting-PLS-DA, combined with NIR spectroscopy, for the non-destructive and accurate origin identification of three kinds of FMHSs (*Gastrodia elata*, *Aurantii Fructus Immaturus*, and *Angelica dahurica*) was proposed. The Boosting-PLS-DA method in this study has the following advantages: (1) the identification ability can be improved with the Boosting-PLS-DA method, for both *Aurantii Fructus Immaturus* and *Angelica dahurica*. The Boosting-PLS-DA identification accuracies of the validation sets and external validation sets with original spectra and all spectral pretreatments were all 100%. However, the accuracies were lower than 99% for the *Aurantii Fructus Immaturus*–DT/SNV-PLS-DA models and *Angelica dahurica*–MSC/SNV-PLS-DA models; (2) the risk of overfitting can be reduced with the ensemble-learning method. Taking the original spectra of *Gastrodia elata* as an example, the accuracies of Boosting-PLS-DA (93.33% for the external validation set) were better than those of PLS-DA (92.22% for the external validation set); (3) the Boosting-PLS-DA method is more suitable for the on-site analysis with the portable instruments, showing high potential for the nondestructive detection of FMHSs. The Boosting-PLS-DA method combines the high robustness of ensemble learning with the strong discriminative capability of discriminant analysis. There are still limitations to this study. The sample sizes of the validation set and external validation set were relatively small. Only three types of FMHS sample data were used to validate the method. The generalizability will be further validated with a sufficiently large external validation set and more types of FMHSs.

## 4. Materials and Methods

### 4.1. Samples

Three kinds of FMHSs (*Gastrodia elata*, *Aurantii Fructus Immaturus*, and *Angelica dahurica*) were purchased from LBX Pharmacy Chain Joint Stock Company, Changsha, China. Each FMHS included samples from three different origins, with 70 samples collected from each source. *Gastrodia elata* samples were collected from Yunnan, Guizhou, and Sichuan. *Aurantii Fructus Immaturus* samples were collected from Sichuan, Jilin, and Jiangxi, while *Angelica dahurica* samples were collected from Sichuan, Zhejiang, and Henan. The Kennard–Stone (KS) algorithm is a widely used dataset-partitioning method [37]. In this study, calibration and validation set samples were obtained using the KS algorithm with a ratio of 4:3 [38]. In addition, to further verify the accuracy and robustness of the model, 30 samples of each origin were obtained as external-validation-set samples one month later.

### 4.2. Instrumentation and Spectra Measurement

Spectra were collected by a portable grating NIR spectrometer (i-Spec Plus, Metrohm, Herisau, Switzerland). After preheating the instrument for 30 min in a dry environment at room temperature (25 °C), the samples were directly placed at the center of the light spot, and spectra were obtained by using the integrating sphere diffuse reflectance mode. The spectral scanning range was 890–1720 nm, with 511 data points.

### 4.3. Data Analysis

There are often interferences, such as peak overlapping, baseline drift, and signal noise in NIR spectra. Spectral pretreatment methods can eliminate the above interferences in spectra to a certain extent. The most widely used pretreatment techniques can be divided into two categories: scatter-correction methods and spectral derivatives. The scatter-correction methods, including SNV and MSC, can be applied to eliminate the influence of solid surface scattering [39]. The derivative algorithms, such as first derivative, second derivative, and CWT, can be used to eliminate background and baseline drift interferences [40]. In addition, the baseline drift interference can be eliminated to a certain extent with DT. Therefore, in this study, six spectral pretreatment methods, including CWT, DT, SNV, MSC, first derivative, and second derivative, were used. In the calculations of the CWT method, the “haar” wavelet and scale = 20 were adopted. Savitzky–Golay derivative was used with a window of 17 and a polynomial order of 2 in the calculations of the first derivative and second derivative.

In the boosting strategy, multiple models are developed according to the distribution of the sample weights obtained and trained iteratively under the guide of the weights. The predictions can be determined by using the weighted median of a confidence indicator. As an ensemble learning method, the boosting strategy can produce higher predictive accuracy combined with partial least squares (PLS) than single model strategy. DA is a statistical strategy that uses category information from datasets to create discriminant boundaries for classification. Similarly to the principle of PLS-DA method, a novel pattern recognition method can be developed by combining Boosting-PLS and DA. In addition, it can be inferred that the boosting strategy combined with PLS-DA can obtain higher identification ability than the traditional PLS-DA method.

Therefore, in this study, a new model for pattern recognition named Boosting-PLS-DA was developed to establish the origin identification models and compared with two existing pattern recognition methods (PCA and PLS-DA). The core mechanism of boosting algorithms involves iterative weight adjustment on training samples. In Boosting-PLS-DA, a calibration subset is generated from the training-set samples using a resampling technique, and uniform initial weights are assigned to all samples. Then, sample weights are adjusted based on the error of the sub-model on the calibration set, and the weight of misclassified samples is increased. Finally, identification is achieved by a weighted combination of the outputs of all sub-models, where sub-models with better performance are assigned higher weights. The number of latent variables (LVs) is a key factor affecting the performance of the PLS-based methods. It is necessary to select an optimal number of LVs for Boosting-PLS-DA and PLS-DA. In this paper, the number of LVs was determined via a Monte Carlo cross-validation (MCCV) with modified Wold’s R criterion.

The programs were performed using MATLAB R2022a (The Mathworks, Natick, MA, USA).

## 5. Conclusions

In this study, a new pattern recognition method named Boosting-PLS-DA was proposed. The non-destructive and accurate origin identification of three kinds of FMHSs (*Gastrodia elata*, *Aurantii Fructus Immaturus*, and *Angelica dahurica*) was achieved based on portable NIR spectroscopy with the developed pattern recognition methods. For the analysis of *Aurantii Fructus Immaturus* and *Angelica dahurica*, 100% accuracy of the validation sets and external validation sets was obtained using the Boosting-PLS-DA models. For the analysis of *Gastrodia elata*, the Boosting-PLS-DA models showed significant improvements in external validation set accuracies compared to the PLS-DA, reducing the risk of overfitting. Therefore, the method proposed in this study can be used to accurately identify the origins of *Gastrodia elata*, *Aurantii Fructus Immaturus*, and *Angelica dahurica* samples. However, it still has some shortcomings that need to be improved, such as the insufficient sample size of the external validation set and the limited variety of FMHSs.

## Data Availability

All related data and methods are presented in this paper and the Supplementary Materials.

## Supplementary Materials

The following supporting information can be downloaded at https://www.mdpi.com/article/10.3390/molecules30173565/s1: The data of spectra of *Gastrodia elata*, *Aurantii Fructus Im-maturus*, and *Angelica dahurica*.
